# Novel Insights into the *Proteus mirabilis* Crystalline Biofilm Using Real-Time Imaging

**DOI:** 10.1371/journal.pone.0141711

**Published:** 2015-10-30

**Authors:** Sandra A. Wilks, Mandy J. Fader, C. William Keevil

**Affiliations:** 1 Centre for Biological Sciences, Faculty of Natural and Environmental Sciences, University of Southampton, Southampton, Hampshire, United Kingdom; 2 Faculty of Health Sciences, University of Southampton, Southampton, Hampshire, United Kingdom; University Hospital of the Albert-Ludwigs-University Freiburg, GERMANY

## Abstract

The long-term use of indwelling catheters results in a high risk from urinary tract infections (UTI) and blockage. Blockages often occur from crystalline deposits, formed as the pH rises due to the action of urease-producing bacteria; the most commonly found species being *Proteus mirabilis*. These crystalline biofilms have been found to develop on all catheter materials with *P*. *mirabilis* attaching to all surfaces and forming encrustations. Previous studies have mainly relied on electron microscopy to describe this process but there remains a lack of understanding into the stages of biofilm formation. Using an advanced light microscopy technique, episcopic differential interference contrast (EDIC) microscopy combined with epifluorescence (EF), we describe a non-destructive, non-contact, real-time imaging method used to track all stages of biofilm development from initial single cell attachment to complex crystalline biofilm formation. Using a simple six-well plate system, attachment of *P*. *mirabilis* (in artificial urine) to sections of silicone and hydrogel latex catheters was tracked over time (up to 24 days). Using EDIC and EF we show how initial attachment occurred in less than 1 h following exposure to *P*. *mirabilis*. This was rapidly followed by an accumulation of an additional material (indicated to be carbohydrate based using lectin staining) and the presence of highly elongated, motile cells. After 24 h exposure, a layer developed above this conditioning film and within 4 days the entire surface (of both catheter materials) was covered with diffuse crystalline deposits with defined crystals embedded. Using three-dimensional image reconstruction software, cells of *P*. *mirabilis* were seen covering the crystal surfaces. EDIC microscopy could resolve these four components of the complex crystalline biofilm and the close relationship between *P*. *mirabilis* and the crystals. This real-time imaging technique permits study of this complex biofilm development with no risk of artefacts due to sample manipulation. A full understanding of the stages and components involved in crystalline encrustation formation will aid in the development of new protocols to manage and ultimately prevent catheter blockage.

## Introduction

The long-term use of indwelling catheters has been shown to result in an almost permanent bacterial colonisation of urine [1]. Stickler [2] illustrated how 25% of patients with short-term catheters (< 7 days) acquired bacteria, with each additional day representing a 5% increase in the likelihood of infection, rising to 100% of patients with long-term catheters (≥ 30 days). Urinary tract infections (UTI) are the second most frequent (17.2%) cause of healthcare associated infection (HCAI) in hospitalised patients in England [3]. However, infection risk is not the only complication of long-term catheterisation; catheters can also regularly become blocked following the formation of crystalline deposits and encrustations.

Up to 50% of patients with long-term catheters will experience encrustations and blockages leading to additional trauma and discomfort, and to high healthcare demands in terms of nursing visits and emergency referrals for catheter replacement [4]. Encrustations are due to the presence of urease-producing bacteria, primarily *Proteus mirabilis* [5, 6]. The role of *P*. *mirabilis* in the production of crystalline biofilms and encrustations has been described previously [7, 8] with urease-production leading to an accumulation of ammonia, which elevates the urine pH causing crystal formation. Crystalline material has been identified as being composed of struvite (magnesium ammonium phosphate) and apatite (calcium phosphate) [9, 10].

Much work has looked at ways to prevent bacterial attachment and subsequent encrustation formation. These have included the use of antibacterial compounds such as the incorporation of silver [11]. However, a large patient study by Pickard *et al*. [12] showed how the use of silver-impregnated catheters had no reduction on UTI prevalence and a smaller study [13] found no effect on blockages. Alternative approaches include incorporation of nitrofurazone [14], electrical currents [15], hydrogel coatings [16] and modulation of urine pH to prevent crystal formation [17]. However, despite such varied solutions, the problem of biofilm formation and persistence remains. There is a continued lack of understanding of catheter-associated biofilms, meaning that a solution is being sought when the nature of the problem remains misunderstood. This is of particular relevance for the complex crystalline biofilms formed by *P*. *mirabilis*.

Previous studies have used either scanning electron microscopy (SEM) [13, 18, 19, 20], environmental SEM (ESEM) [21] or scanning laser confocal microscopy (SLCM) [22, 23] to visualise biofilms on urinary catheters. Both of these imaging techniques have a number of inherent limitations. In the majority of cases, SEM, although providing high magnification and high contrast imaging, requires stringent sample dehydration as analysis is carried out under vacuum. This can lead to artefacts, which can distort results. This is particularly evident when imaging biofilms where large amounts of exopolymeric substances (EPS) are present and whose structure can be severely distorted during this process. Low vacuum ESEM can offer a suitable alternative [21] but requires highly specialised equipment and, in some cases, additional sample preparation procedures due to short focal distances or poor contrast resolution, resulting in the need for fixation. Jones *et al*. [22] used SLCM, which provides a three-dimensional map of the biofilm without the need for such destructive preparation. The limitations of this are that the sample must fluoresce, meaning that, in most cases, bacteria and associated structures require labelling. This also limits the understanding of interactions between the bacteria and the catheter material.

In the current study, a non-contact, non-destructive real-time imaging method has been used to study the attachment and biofilm development of *P*. *mirabilis* on two commonly used catheter materials over a 24 day time period to understand the stages of crystalline biofilm development. Episcopic differential interference contrast (EDIC) microscopy allows direct analysis of biofilms on curved and solid materials, using long working distance objectives and episcopic illumination to create a pseudo-3D image with no need for sample preparation [24].

## Materials and Methods

Foley catheters (12 ch) made of two commonly used materials (100% silicone–Rüsch; hydrogel latex–Bard) were cut into 1 cm lengths horizontally and longitudinally, using sterile fine point scissors. These sections were stored in sterile Universal tubes until use. Artificial urine was prepared as described in Brooks & Keevil [25] and filter-sterilised. Two sections of the each catheter were placed into each well of a six-well tissue culture plate, with a single well representing one time point. A 5 ml volume of artificial urine was added to each and care was taken to ensure the catheter sections were well covered. As an inoculum, an overnight urine broth culture of ureolytic *P*. *mirabilis* NCTC 10975 was centrifuged at 7500 rpm for 10 min and the supernatant discarded. The pellet was resuspended in the same volume of artificial urine and vortex mixed to disperse the cells. A 100 μl aliquot was added to each well (giving a final concentration of 10^9^ cfu ml^-1^). The tissue culture plates were covered and incubated in a 37°C incubator.

Catheter sections were removed after 1, 2, 4, 6 and 24 h, and then at each subsequent 24 h time period over a total of 24 days. Every 24 h, contaminated artificial urine was removed from each well and replaced with 5 ml fresh medium. Each catheter section was removed using sterile forceps, gently immersed in sterile water to remove any planktonic cells and placed in covered petri dishes. These were stored in a humid environment at 4°C prior to imaging. One catheter section was stained with a combination of 4',6-diamidino-2-phenylindole (DAPI) and concanavalin A (ConA). DAPI was prepared as a stock solution with a concentration of 1 mg ml^-1^ which was stored at -20°C. A working solution of 10 μg ml^-1^ was prepared in sterile distilled water and applied to the lumen of the catheter section and incubated at room temperature for 15 min in the dark. Following this incubation, excess stain was removed and the section gently rinsed with sterile distilled water. Tetramethylrhodamine (TRITC)-labelled ConA was added at a concentration of 50 μg ml^-1^, covering the catheter lumen, and incubated at room temperature for 5 min in the dark. Excess ConA was removed and each section gently washed with sterile distilled water. The sections were air dried and stored in the dark in a humid environment at 4°C.

The catheter sections were examined using an episcopic differential interference contrast (EDIC)/epifluorescence (EF) Nikon Eclipse LV100D microscope (Best Scientific, UK) [24] using a metal halide light source (EXFO X-CITE 120 fluorescence system), long working distance metallurgical objectives (Nikon Plan Achromat) and a high resolution camera (QImaging Retiga EXi Cooled Digital CCD monochrome camera with RGB colour filter module). Images were captured and processed using ImagePro image capture software. Experiments were repeated three times with two sections (from each experiment) used for EDIC microscopy at each time point. The entire length of the sections was examined using a x 50 objective with representative images being taken over 10–50 fields of view (including at the higher magnification of x 1000).

## Results

### EDIC microscopy on unused catheters

**Fig 1:**
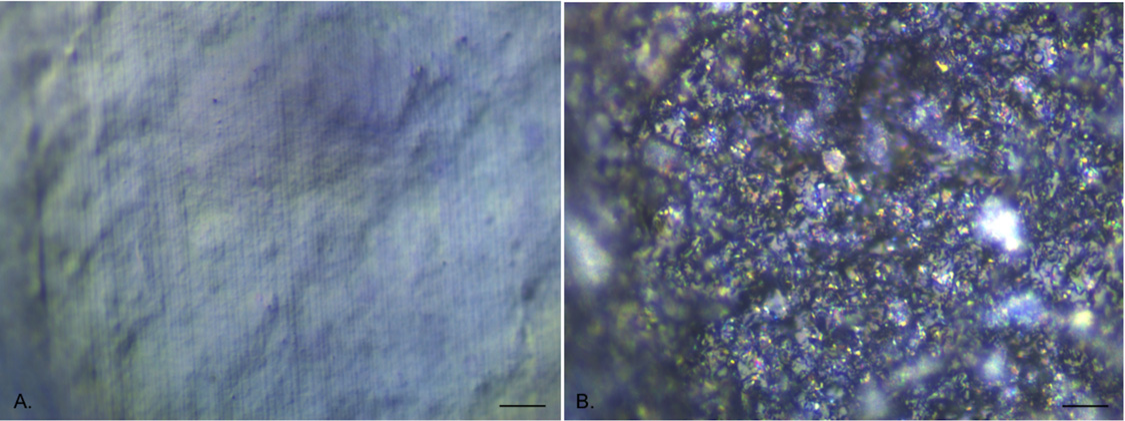
EDIC images of unused catheter sections showing surface topography. a. All silicone catheter. b. Hydrogel latex catheter. (Magnification x 1000, bar = 10 μm).

EDIC microscopy was found to be effective at imaging the surface of both silicone and hydrogel latex catheters, with the long working distance objectives allowing for the curved surface of the internal lumen (Fig 1). The all silicone catheter surface (Fig 1A) was considerably smoother than the hydrogel latex but still had larger scale undulations, areas of pitting and parallel longitudinal striations resulting from the manufacturing process; all providing potential attachment sites for bacteria. In contrast, the hydrogel latex catheter (Fig 1B) had a highly disordered surface topography, appearing very rough and being composed of different particle types, as seen by changes in reflective properties.

### *P*. *mirabilis* attachment to silicone catheters

**Fig 2:**
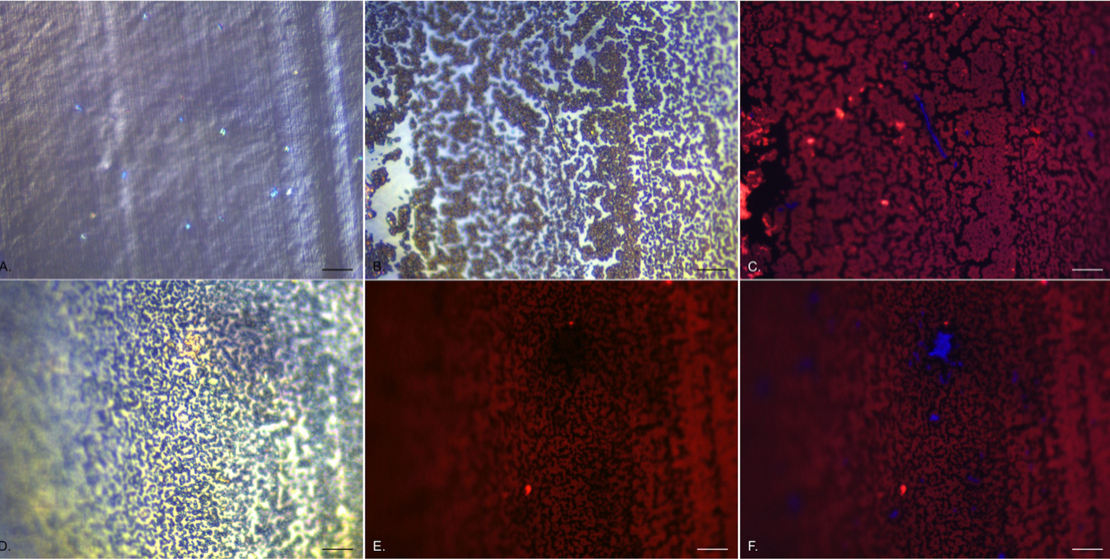
Early attachment events of *P*. *mirabilis* to silicone catheter sections. a. EDIC/DAPI composite image showing catheter surface and individual, attached bacteria after 1 h exposure. b. EDIC image after 2 h exposure, showing accumulation of extracellular material. c. corresponding DAPI/ConA composite image showing clearly labelled elongated swarmer cells and ConA labelled material. d—f. 4 h exposure. d. EDIC image showing extensive deposition of extracellular material and visible clusters of bacteria. e. ConA labelled material showing areas with no staining which correspond to DAPI labelled clusters of bacteria as seen in f. DAPI/ConA composite image. (Magnification x 1000, bar = 10 μm).

In the first 1 h of exposure of the catheter sections to *Proteus*-contaminated artificial urine, there were increasing numbers of individual cells attaching to the surface. By culture analysis (after removing attached bacteria by vortexing with glass beads), it was found that approximately 1.5 x 10^4^ colony forming units (cfu) cm^-1^ length were attached following 15 min exposure. This increased to approximately 3 x 10^6^ cfu cm^-1^ after 2 h exposure (Wilks, unpublished data). These were distributed over the entire surface area of the catheter sections, with individual bacteria being strongly labelled by DAPI. Once labelled with DAPI, it was also possible to locate the bacteria using EDIC only (Fig 2A) as the pseudo three-dimensional imaging from EDIC illumination made them clearly distinguishable from the silicone surface. After 2 h exposure, these individual cells were surrounded by large amounts of additional material, which had a different imaging structure when observed under EDIC (Fig 2B). When dual stained with DAPI and ConA, it was possible to see individual DAPI-labelled bacteria surrounded by ConA-labelled material (Fig 2C). The majority of the DAPI-labelled *P*. *mirabilis* remained approximately 1 μm long but occasional highly elongated cells could be seen, extending up to 40 μm in length (Fig 2C) (these represented less than 1% of the *P*. *mirabilis* population). These could also be identified under EDIC imaging only, with both non-elongated and elongated bacteria demonstrating a highly reflective structure compared to the other material (Fig 2B). This material increased with increasing exposure time, with increasing numbers of *P*. *mirabilis* also attaching and with clusters of bacteria starting to form (Fig 2D–2E) following 4 h exposure. Using EDIC imaging it was possible to see areas where this followed the manufacturing striations of the silicone and the clumping of *P*. *mirabilis*, appearing increasingly more reflective. When dual-labelled, it was found that DAPI-stained bacteria did not also show ConA labelling as evident in Fig 2E where, compared to the composite image in Fig 2F, there was no labelling where clusters of DAPI-labelled cells were located.

**Fig 3:**
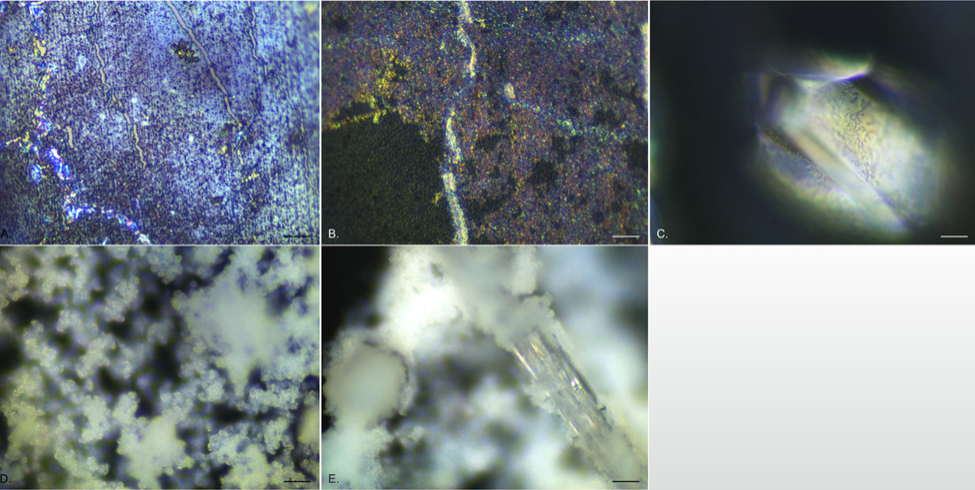
Representative EDIC images showing crystalline biofilm development on all silicone catheter sections exposed to *P*. *mirabilis*. a. 24 h exposure showing multi-layered appearance and highly reflective, motile *P*. *mirabilis*. b. 3 days exposure showing development of a microcrystalline layer attaching to the initial conditioning film below. c. an individual struvite crystal formed after 3 days exposure. d. after 4 days exposure, copious amounts of diffuse crystalline material (apatite) formed, creating a thick three-dimensional structure. e. at the same time (and over the remaining time course), large, rod-shaped crystal embedded in diffuse crystalline material formed, extending in length to 10 mm. (Magnification a—d x 1000, bar = 10 μm; e x 500, bar = 20 μm).

After 24 h, the biofilm started to show a multi-layered appearance (Fig 3A) with areas of dense colonisation separated by regions of more disperse coverage, with channels running through where the catheter surface could be seen. Lines of highly reflective bacteria were observed, often following the channels of exposed catheter or forming clumped groupings (Fig 3A). Within days 2 to 3, another structural layer could be observed, with sheets of a reflective, microcrystalline material covering large areas but clearly laying over the initial biofilm (Fig 3B). In addition, individual single crystals could be found (Fig 3C). After 4 days exposure, a dramatic change was observed with copious amounts of diffuse crystalline material appearing (Fig 3D). This material followed the distribution pattern of the other layers with areas revealing the original catheter surface remaining uncovered. Embedded within this were large crystals, many formed as long (5–10 mm) rods, extending out from the catheter surface. These defined crystals showed a strong association with the more diffuse material, with the latter often wrapping around the crystal edges (Fig 3E). The amount of diffuse crystalline material and defined crystals increased with increasing exposure time, over the 24 days of the experiment, creating a highly three-dimensional structure with clearly distinct layers.

It was not possible to continue to stain bacteria using the DAPI/ConA labelling system as the crystals (both diffuse and defined) adsorbed the fluorescent dyes making it impossible to visualise individual cells. However, using EDIC only, it was possible to see bacteria covering the surface of defined crystals, indicating that they remained present throughout the complex crystalline biofilm (Fig 3C).

**Fig 4:**
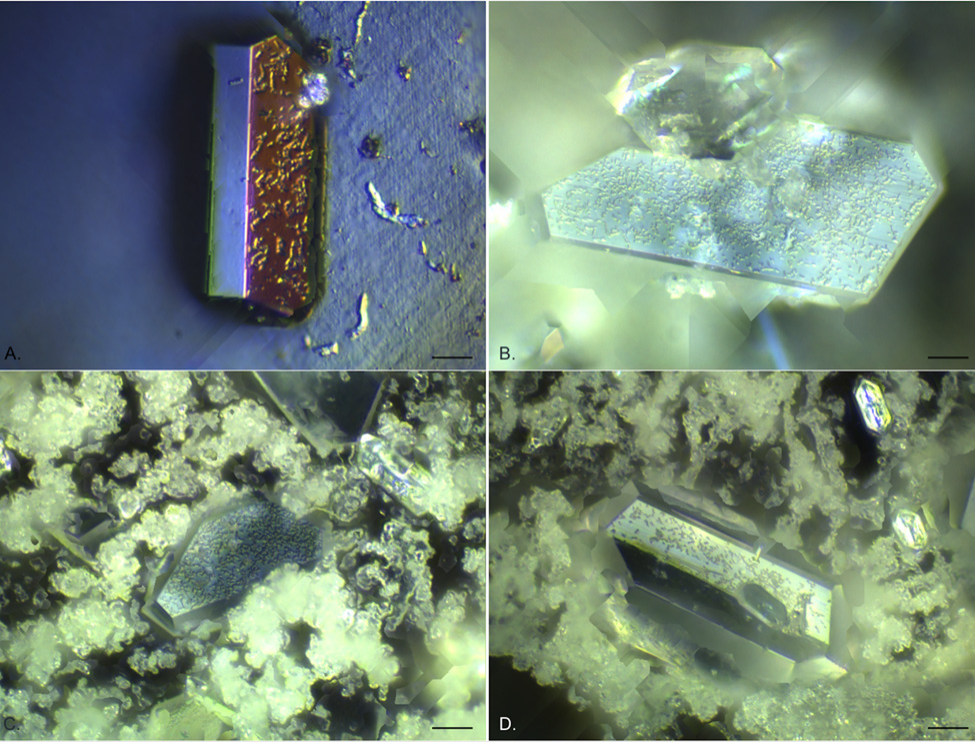
Extended depth of field composite EDIC images of silicone catheter sections following long term exposure to *P*. *mirabilis*. a. 12 days exposure, a single struvite crystal on exposed silicone catheter (where the diffuse crystalline material has detached) with motile *P*. *mirabilis* on the crystal and on the uncolonised surface. b—d. struvite crystals embedded in diffuse crystalline material. b. 15 days exposure. c. 18 days exposure. d. 20 days exposure. (Magnification a x 1000, bar = 10 μm; b—d x 500, bar = 20 μm).

The image processing software allowed for extended depth of field images to be generated by combining a number (generally 100–200) of z-scan fields of view and then optically flattening them, to allow creation of a single image looking through the complex biofilm structure. This allowed further description of the component layers of the biofilms and associated crystals as seen in the examples given in Fig 4. Fig 4A shows an individual struvite crystal on an exposed silicone catheter surface where the diffuse microcrystalline material had been dislodged. Under EDIC illumination it was possible to see highly motile, elongated *P*. *mirabilis* on the catheter surface and clusters of bacteria covering the crystal and spreading over the most extended side. This pattern of crystal colonisation was also seen in crystals embedded amongst the diffuse crystalline material (likely to be apatite), with high numbers of bacteria evident on the crystals as they extended out from the crystal surface (Fig 4B–4D). These crystals continued to develop over the entire time course of the experiment.

### *P*. *mirabilis* attachment to hydrogel latex catheters

It was not possible to use the DAPI/ConA combined staining protocol as the hydrogel latex material exhibited strong autofluorescence in all channels prior to staining. In addition, the highly disordered structure of the hydrogel latex surface (Fig 1B) made imaging of initial attachment events difficult and it was not until longer exposure times that the biofilm development could be observed using EDIC microscopy.

**Fig 5:**
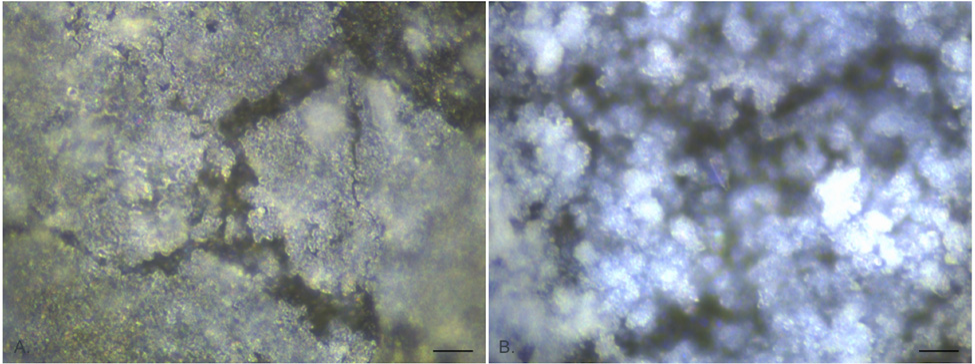
Representative EDIC images of biofilm development on hydrogel latex catheter sections. a. 3 days exposure showing thick microcrystalline layer. b. 9 days exposure showing development of a three-dimensional, diffuse crystalline structure, extending out from the surface. (Magnification x 1000, bar = 10 μm).

**Fig 6:**
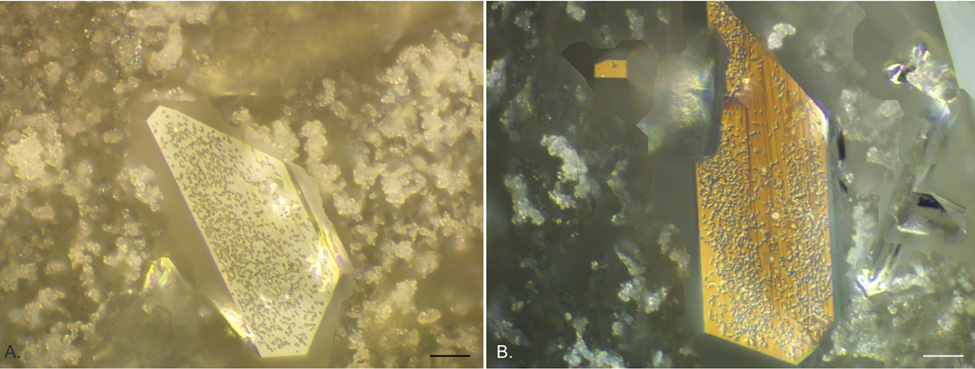
Representative extended depth of field composite EDIC images of hydrogel latex catheter sections following long term exposure to *P*. *mirabilis*. Struvite crystals embedded in diffuse crystalline material. a. 10 days exposure. b. 15 days exposure. (Magnification x 500, bar = 20 μm).

In the first 2 days of exposure to *P*. *mirabilis*, it appeared that diffuse material was collecting on the surface but this was not consistent across samples. However, after 3 days exposure, clear sheets of a microcrystalline material could be seen (Fig 5A) above the catheter material, separated by channels and starting to show further development of material extending from them. This material had a similar, but denser, composition to that observed for silicone catheters after 3 days (Fig 3B). This was accompanied by the presence of occasional defined crystals. The crystalline material continued to accumulate over subsequent days until it covered the entire catheter surface making it no longer possible to see the material structure (Fig 5B). Copious amounts of this diffuse crystalline material extended several hundreds of microns. Once formed, this continued to accumulate over the entire experimental time course (24 days). Using the extended depth of field function, z-scan images through the material were possible showing the complex layering of the system and the different component structures (representative images are shown in Fig 6A and 6B). It was also clear to see large numbers of *P*. *mirabilis* attached to the surface of the defined crystals, illustrating again the close relationship between the bacteria and the crystalline structures.

## Discussion

**Fig 7:**
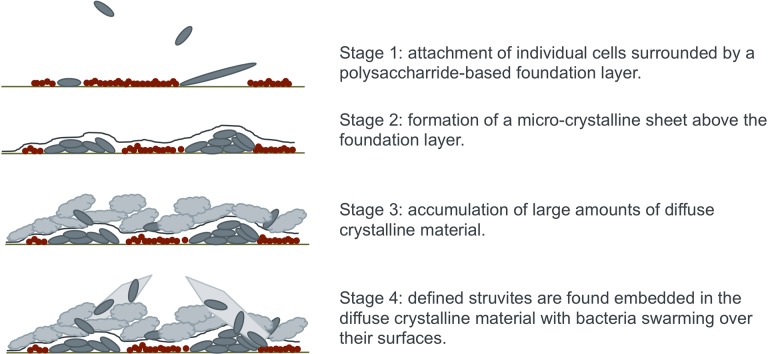
Schematic representation of the four stages of crystalline biofilm development by *P*. *mirabilis*. This shows the development from initial conditioning film to complex crystalline biofilm with *P*. *mirabilis* found throughout the structure.

Using EDIC microscopy, the complex crystalline biofilm formed by the presence of urease-producing *P*. *mirabilis* can now be defined as having four distinct layers or components (as shown schematically in Fig 7). In summary, these are: (1) an initial conditioning film formed from low numbers of primary colonising bacteria and large amounts of sugar-based carbohydrates, (2) a sheet-like microcrystalline material which covers this conditioning film and from which (3) copious amounts of diffuse crystalline material extends with (4) embedded, defined crystals covered in highly motile *P*. *mirabilis*. This greatly furthers our understanding of the complexity of the *P*. *mirabilis*-mediated crystalline biofilm.

In order to find ways to control and prevent encrustations, it is essential that we have a full understanding of biofilm formation. The observations made in this work advance our understanding when placed in the context of previous studies. When considering the various stages, it is clear that if primary attachment and development of a conditioning film can be prevented the subsequent formation of encrustations could have reduced impact.

Our findings support observations made in some previous studies [2, 7, 8, 13, 17, 19, 21, 22, 26, 27] but also adds further important information. The use of EDIC microscopy has been shown to be a rapid and effective method for visualising crystalline biofilm development directly on catheters, with no need for destructive sample preparation procedures. This technique offers considerable improvement over high vacuum SEM, where samples must undergo dehydration leading to artefacts. The use of ESEM removes the need for a high vacuum environment, provides high magnification imaging and can allow concurrent analyses such as x-ray spectroscopy [21]. However, it does require highly specialised equipment and can be limited by focal length unless samples are manipulated. EDIC microscopy offers increased flexibility by being able to be combined with epifluorescence, allowing the use of a range of biomarkers and indicators, and is able to image a range of solid, three-dimensional materials (including catheters) directly [24]. SLCM has also been used previously [22, 23] and while this does not result in disruption of the biofilm due to the need for a fluorescent signal, this method provides only limited information on the interaction with the catheter surface and crystalline material.

The current study has used a static system for biofilm formation on the catheters but the observations follow the trends described previously where a bladder model has been used [8, 13, 17, 18, 19, 26]. The use of a physiologically correct artificial urine [24] is important as previous studies have demonstrated the changes in biofilm formation when a nutritious broth media was used [22, 23]. Some studies [13] have used pooled human urine and while this could be argued to be more realistic, by using a defined artificial urine medium, we have a known composition, meaning observations are consistent across samples. The system used here provides a generalised model of *P*. *mirabilis* crystalline biofilm formation under very controlled conditions. Using this model, it is then possible to vary each condition and examine the effects on biofilm formation.

Our findings differ from those of previous researchers. Stickler & Morgan [26] found, using a bladder model, that the first layer was composed of microcrystalline deposits (identified by SEM) and formed within 1–2 h around the eye-hole area of all silicone catheters. The microcrystalline characteristics of this initial layer were only confirmed by x-ray analysis after a longer exposure of 4 h. They went on to indicate that bacteria preferentially colonise this material forming microcolonies and leading to biofilm development. However, observations were also made that the cells did occasionally attach to areas where this material was absent implying that the attachment process was not dependent on crystal formation (although this was preferred). Winters *et al*. [27] and Morris *et al*. [8], following analysis by SEM, also suggested that the colonisation of crystalline deposits by bacteria is the first stage of *P*. *mirabilis* biofilm development. An alternative to the preliminary microcrystalline layer was made by Stickler [28] who proposed that the initial conditioning film could also be formed of host proteins that allow attachment of bacteria via fimbriae. In a laboratory study using an artificial urine medium, such as reported here, host proteins would not be present.

In the current work, evidence of a rapidly forming foundation or conditioning layer was present and within the same time period of 1–2 h. It was also possible to locate individual *P*. *mirabilis* using DAPI labelling and by EDIC imaging alone. Attachment of these cells occurred prior to the formation of the foundation layer directly onto the catheter surface. As the initial layer formed, occasional highly elongated, motile cells were observed followed by the formation of clusters of bacteria. Previously, these have been described as microcolonies but may instead represent the coordinated motility of swarming *P*. *mirabilis* which has been shown to form multicellular rafts [29]. Such multicellular rafts were observed when *P*. *mirabilis* was grown in LB medium and exposed to a surface. There have been suggestions that this can happen in urine but this has not been directly shown. The appearance of elongated cells follows observations made by Jones *et al*. [22] where such highly motile bacteria were found extending out from the biofilm and were considered to be primary colonisers of the surface. When labelled with TRITC ConA, these elongated cells and clusters were not visible but the foundation layer material gave a strong signal. This is indicative of polysaccharide elements; common constituents of biofilm extra polymeric substances (EPS). Several studies [13, 26, 28] refer to *P*. *mirabilis* as colonising this foundation layer, however, dual labeling with DAPI and TRITC ConA clearly shows that the DAPI-labeled bacteria were negative for ConA. It is apparent that the area around the cells also remained unlabeled. This indicates that these primary colonisers were attaching directly to the catheter surface, with the foundation layer forming around them.

As exposure time increased, this foundation layer took on a multidimensional appearance interspersed with tracks of highly reflective bacteria running throughout the system; the highly reflective structure of these *P*. *mirabilis* could indicate a direct relationship with crystalline formation. Such observations have not been made using SEM, ESEM or SLCM. After 4–6 h exposure to *P*. *mirabilis*, the rapid formation of a crystalline 'sheet-like' layer was observed and staining with ConA and DAPI could no longer be used. This was due to all material present adsorbing the stains and this continued over the entire course of the experiment. This time frame correlates with observations made by Stickler and Morgan [26]. The formation of a distinct microcrystalline layer has only been described previously by Holling *et al* [21] where ESEM was used to examine *P*. *mirabilis* crystalline biofilms formed on catheters using a bladder model and an artificial urine medium. Two distinct crystal types were observed, with type 2 being described as flat, 'sheet-like' material. Using EDIC microscopy, we have been able to show how this material extends over large areas of the foundation layer and is formed in advance of the bulk crystalline components.

In the current study, after a further 24 h exposure the entire surface was covered with diffuse crystalline material. The formation of large single crystals wrapped in this material followed shortly after. These last two phases were observed in both silicone and hydrogel latex catheters, occurring in quick succession in the biofilm development. EDIC microscopy showed *P*. *mirabilis* to be integrated within the diffuse crystalline material and found to be covering individual crystals.

Previous studies have identified the majority of crystalline components to be apatite (calcium phosphate) (including the 'sheet-like' layer) and struvite (magnesium ammonium phosphate) [9, 10, 21]. Stickler & Morgan [26] showed copious amounts of diffuse crystalline material to be identified as apatite and demonstrated the presence of coffin shaped struvites. Other studies [29, 30] found apatite to be precipitated from urine prior to struvite formation. These two crystalline materials can be clearly observed under SEM although it is likely that some would be lost during sample preparation as the apatite, in particular, is only loosely bonded to the surface and can be easily removed once dry. By using the non-contact, non-destructive EDIC microscopy technique, we have been able to study these materials with no risk of structural disruption. Combining this with an ability to image individual bacteria and additional components of the EPS such as the polysaccharide conditioning film, provides an important new understanding of the complex nature of this biofilm.

In addition, EDIC microscopy has allowed more detailed assessment of the interactions between bacterial attachment and the catheter surface. The surfaces of both all silicone and hydrogel latex catheters show numerous potential attachment sites, with the latter exhibiting a highly disordered topographical structure. Previous studies [26, 28] have commented on material roughness but suggested primary attachment sites to be around the eye holes and, in the case of hydrogel latex, attached to diatom skeletons embedded within the material. In the current study, striations running the length of silicone catheters formed during manufacturing, were often followed by initial development of the conditioning film. The highly disordered surface of hydrogel latex catheters made imaging initial attachment events impossible but as the crystal formation began, it was possible to distinguish the various components of the crystalline biofilm. Tracking biofilm development by EDIC microscopy has not only shown that crystalline biofilm occurs equally on silicone and hydrogel latex but that the two materials had no effect on the time progression of development.

This study has described the formation of biofilms in urinary catheters by *P*. *mirabilis*, using non-destructive methods revealing four phases of biofilm development. New components and processes involved in biofilm development have been identified, challenging traditional theories and previous findings. This work shows a clear time-dependent sequence to crystalline biofilm formation and by understanding these stages of development, new targets in the search for a biofilm resistant catheter material or treatment protocol may be identified. A solution to this serious healthcare issue is long overdue but further insight into the nature of the crystalline biofilm is essential.
